# Cumulative Sulfate Loads Shift Porewater to Sulfidic Conditions in Freshwater Wetland Sediment

**DOI:** 10.1002/etc.4410

**Published:** 2019-05-27

**Authors:** Nathan W. Johnson, John Pastor, Edward B. Swain

**Affiliations:** ^1^ Department of Civil Engineering University of Minnesota Duluth Duluth Minnesota USA; ^2^ Department of Biology University of Minnesota Duluth Duluth Minnesota USA; ^3^ Minnesota Pollution Control Agency St. Paul Minnesota USA

**Keywords:** Sulfide, Porewater, Wetland sediment, Rooting zone, Wild rice

## Abstract

It is well established that sulfide can be toxic to rooted aquatic plants. However, a detailed description of the effects of cumulative sulfate loads on sulfide and iron (Fe) porewater geochemistry, plant exposure, and ecological response is lacking. Over 4 yr, we experimentally manipulated sulfate loads to self‐perpetuating wild rice (*Zizania palustris*) populations and monitored increases in the ratio of sulfur (S) to Fe in sediment across a range of sulfide loading rates driven by overlying water sulfate. Because natural settings are complicated by ongoing Fe and S loads from surface and groundwater, this experimental setting provides a tractable system to describe the impacts of increased S loading on Fe–S porewater geochemistry. In the experimental mesocosms, the rate of sulfide accumulation in bulk sediment increased linearly with overlying water sulfate concentration up to 300 µg‐SO_4_ cm^–3^. Seedling survival at the beginning of the annual life cycle and seed mass and maturation at the end of the annual life cycle all decreased at porewater sulfide concentrations between 0.4 and 0.7 µg cm^–3^. Changes to porewater sulfide, plant emergence, and plant nutrient uptake during seed production were closely related to the ratio of S to Fe in sediment. A mass balance analysis showed that porewater sulfide remained a small and relatively transient phase compared to sulfate in the overlying water and Fe in the sediment solid phase. The results illuminate the evolution of the geochemical setting and timescales over which 4 yr of cumulative sulfate loading resulted in a wholesale shift from Fe‐dominated to sulfide‐dominated porewater chemistry. This shift was accompanied by detrimental effects to, and eventual extirpation of, self‐perpetuating wild rice populations. *Environ Toxicol Chem* 2019;38:1231–1244. © 2019 The Authors. Environmental Toxicology and Chemistry published by Wiley Periodicals, Inc. on behalf of SETAC.

## INTRODUCTION

The introduction of sulfate to freshwater ecosystems can alter the processing of nitrogen (N), phosphorus (P), iron (Fe), mercury, carbon (C), and other elements (Ardon et al. 2013; Myrbo et al. 2017b), thereby enhancing eutrophication (Lamers et al. 1998) and greenhouse gas emissions (Helton et al. 2014), changing contaminant transformations and mobility (Besser et al. 1996; Jeremiason et al. 2006) and the life cycles of vegetative and benthic organisms (Wang and Chapman 1999; Lamers et al. 2013; Hopfensperger et al. 2014). Because sulfate is a terminal electron acceptor capable of driving heterotrophic anaerobic microbial metabolism (Weston et al. 2011; Myrbo et al. 2017b), rooted freshwater plants and other sediment‐dwelling organisms are affected when sulfate is converted to sulfide in anoxic sediments (Bagarinao 1992; Lamers et al. 2013; Pastor et al. 2017). Changes in sulfate loads to freshwater ecosystems occur because of hydrologic modification in coastal zones, increased atmospheric sulfur (S), municipal and industrial wastewater inputs, and differences in wetting/drying cycles induced by climate change (Driscoll et al. 2001; Craft et al. 2009; Hao et al. 2014; Schoepfer et al. 2014). These changed sulfate loads have had ecological consequences including shifts in vegetation, accelerated eutrophication, toxicity to benthic organisms, increased organic matter mineralization in sediment, and increased mercury methylation (Lamers et al. 2002; Smolders et al. 2003; Li et al. 2009; Neubauer 2013; Kinsman‐Costello et al. 2015; Myrbo et al. 2017b).

In wetland plants, dissolved sulfide interrupts metalloenzymes in the electron transport chain and disrupts the ability to take up nutrients (Joshi et al. 1975; Koch et al. 1990; Martin and Maricle 2015). In contrast, solid‐phase sulfides are inert and nontoxic to organisms (Morse et al. 2007). The extent to which microbial sulfate reduction can increase sulfide concentrations in sediment porewaters to toxic levels depends, in part, on the quantity of reactive metals available to precipitate with sulfide, thus removing sulfide from porewaters (Wang and Chapman 1999; Heijs et al. 1999; Pollman et al. 2017). Iron, often the most abundant metal in aquatic sediment, has been identified as having an ameliorating effect on the impacts of sulfide loading to aquatic plants in both experimental (Van der Welle et al. 2006) and natural (Schoepfer et al. 2014) settings. The amount of sulfide in sediment, relative to the quantity of Fe, has been termed “degree of sulfidation”, and this framework has been used extensively to understand the current and paleolimnological implications of S and Fe cycling (Morse and Luther 1999; Wijsman et al. 2001). More recently, Schoepfer et al. (2014), van der Welle et al. (2007), and Julian et al. (2017) have studied the balance of Fe and S loads specifically in freshwater systems impacted by sulfate from agricultural pollution and seawater intrusion.

Hydroponic and short‐term mesocosm experiments, involving the direct (and often continuous) injection of sulfide or Fe to aquatic media or sediment (compiled in Lamers et al. 2013), have been widely used to identify mechanisms of sulfide's toxicity to plants (Koch and Mendelssohn 1989; Martin and Maricle 2015). However, these typically short‐term experiments are not conducive to examining natural processes related to coupled Fe and S cycling that occur during natural loading to intact aquatic sediment. Several field studies at sulfate‐impacted freshwater systems have interpreted the vegetative and in situ porewater chemistry implications of S loading in the framework of degree of sulfidation (Burton et al. 2006; Morse et al. 2007; Hopfensperger et al. 2014; Schoepfer et al. 2014; Julian et al. 2017). Studies in natural settings, however, typically cannot control conditions in a manner conducive to a detailed evaluation of evolving sediment sulfide in response to altered sulfate loading. Long‐term (e.g., multiyear) mesocosm studies employing a slow and continuous production of sulfide in sediments (Lamers et al. 1998; Howes et al. 2005; van der Welle et al. 2006; Geurts et al. 2009) are most conducive to investigating the progressive depletion of reactive Fe in a setting analogous to natural wetlands with enhanced sulfate loads. No studies of this nature, however, have described in detail the evolution of toxic, sulfide‐rich, geochemical conditions in porewater over the entire timescale needed for increased sulfide production to overwhelm the loading of Fe to sediment in a natural setting.

The objective of the present study was to test the impacts of sulfate loading on Fe geochemistry and the population dynamics of an annual freshwater wetland species, wild rice (*Zizania palustris* L.). Wild rice grows in thick monotypic stands mostly confined to shallow lakes and rivers of the Lake Superior region in North America (Day and Lee 1990) and is usually found in waters with sulfate concentrations <10 mg L^–1^ (Moyle 1944). Sulfate‐poor freshwater ecosystems have experienced increases in surface water sulfate in response to elevated rainwater sulfate over the past century (Driscoll et al. 2001). More recently, wild rice and other freshwater aquatic plants have become threatened by increases to sulfate loading from agricultural and industrial discharges (Lens et al. 1998; Orem et al. 2011; Berndt et al. 2016), which can elevate surface water sulfate significantly above natural levels and those elevated from acid rain. Hydroponic, mesocosm, and field results have shown that porewater sulfide is toxic to wild rice at levels near 0.3 µg cm^–3^ (Pastor et al. 2017) and that sediment Fe is a key factor controlling the presence of wild rice in field conditions (Myrbo et al. 2017a; Pollman et al. 2017). However, the evolution of sediment geochemistry in response to altered S loads has not been described in detail. The present study was conducted in outdoor mesocosms containing self‐perpetuating wild rice populations growing in natural wild rice sediment. The experimental design allowed for a precise manipulation of the loading rate of S to continuously inundated sediment by controlled concentrations of sulfate in the overlying water (Pastor et al. 2017). The temporal and spatial evolution of porewater geochemistry, solid‐phase geochemistry, and impacts to the self‐perpetuating wild rice populations were quantified over 4 yr. The present results interpret how the interannual increases in porewater sulfide in mesocosm sediment correspond to the loading of sulfate from surface water and contextualize the geochemical setting in which detrimental effects to, and eventually extirpation of, wild rice was observed.

## METHODS

Sulfate amendments to the overlying water of wild rice tank mesocosms described by Pastor et al. (2017) began in the summer of 2011 and continued through 2015 at 5 different amendment levels: nominally approximately 0, 50, 100, 150, and 300 mg‐SO_4_ L^–1^. This range is well above that found in remote regional streams impacted only by atmospheric S sources but spans that found in Minnesota surface waters (Myrbo et al. 2017b), some of which are impacted by neutral pH mining‐influenced water (Berndt et al. 2016). This range of sulfate also encompasses the US Environmental Protection Agency's (USEPA's) 250 mg L^–1^ secondary drinking water standard and the existing 10 mg L^–1^ standard to protect wild rice in the state of Minnesota (Myrbo et al. 2017a). Methods of maintaining the mesocosms are described in detail by Pastor et al. (2017). Briefly, 6 replicate tanks at 5 sulfate amendment levels were maintained using polyethylene stock tanks (0.75 m^2^ area, 61 cm depth) containing a 10‐cm‐thick layer of sediment rich in organic matter (15% C, 1.1% N, 1.5% Fe, 0.005% S) from a local wild rice water body and a 23‐cm‐deep overlying water column. Water and sulfate levels in mesocosms were maintained by weekly additions of well water averaging 8 to 10 mg L^–1^ sulfate, 0.17 mg L^–1^ Fe, and <0.05 mg L^–1^ N and P. When necessary (approximately every 2 wk during the growing season), sodium sulfate stock solution was added to each of the 30 experimental mesocosms to account for rain water dilution, evaporation, and flux of sulfate into sediment. The exact concentrations of overlying water in sulfate‐amended tanks are slightly less than the targeted nominal concentrations because of rainfall and other factors, whereas the overlying water of control tanks (no extra sulfate addition) averaged approximately 8 mg/L because of sulfate in well water. The exact overlying water sulfate concentrations over the 4 yr are reported in Pastor et al. (2017).

### Sample collection

During each of the summers from 2012 to 2015, passive porewater equilibrators (peepers) were deployed to make depth‐profile porewater measurements in mesocosms. During the summer of 2012 and 2013, peepers were deployed in 2 mesocosms at each of 4 sulfate treatment levels (control, 50, 150, and 300 mg L^–1^). In 2014 and 2015, peepers were deployed in to mesocosms at all 5 treatment levels. During each of the 4 experimental yr, depth profiles of porewater chemistry were collected with peepers in early summer (June) and late summer (August). Sediment cores were collected in 2013 and 2015 during peeper retrieval in locations coincident with peepers, sectioned into 1‐ to 3‐cm intervals, and frozen for preservation. Cores were collected with a 2.5‐cm‐diameter, thin‐walled polycarbonate manual piston corer to minimize disturbance and preserve sediment structure during collection. Cores and porewater sampling locations were targeted to remain at least 5 to 10 cm away from wild rice stems and roots.

Peepers similar to those described in Teasdale et al. (1995) were constructed of 0.5‐inch‐thick, 24 × 6–inch acrylic sheet plastic beveled at one end and milled to contain 35 wells that were 1 cm deep and spaced 1.56 cm apart. Large‐diameter (~12 inch) circular filter paper (0.45 µm polyethersulfone; Supor; Pall Life Sciences) and a protective nylon mesh (200‐µm openings; Industrial Netting) were cut to fit over the wells and secured in place using a face plate with openings corresponding to each well. Small stainless steel machine screws were used to seal the face plate tightly against the filter paper and prevent water from bypassing filter material. Peepers were assembled in the laboratory while submerged in distilled water to avoid any bubbles. They were then immediately placed in an upright container filled with distilled water that was purged of oxygen by a continuous flow of N through a fine bubble diffuser. After 4 to 6 d, the peepers were transported to the field mesocosms in deoxygenated water, inserted into the sediment, and secured to the edge of the mesocosms with nylon string. Following a 2‐ to 3‐wk deployment (Fisher and Reddy 2001), peepers were removed from the sediment and submerged in water purged of oxygen during the brief (<2 min) transport to a processing station. Mud was quickly removed from the filter paper surface with distilled water, and the tops of wells were quickly dried with Kimwipes before the peeper was placed into a N‐filled bag for sample extraction (Koretsky et al. 2008). Beginning with the deepest well, samples were extracted from each peeper by puncturing the N‐filled bag, nylon mesh, and filter paper with a hypodermic needle affixed to a polypropylene syringe barrel. The extracted sample was immediately injected into vials preloaded with reagents for Fe and sulfide analysis. pH was measured immediately (within 30 s) on a separate sample aliquot prior to degassing of carbon dioxide from the sample. A final aliquot of porewater sample for anion analysis was filtered through a cation exchange filter (Dionex; OnGuard II‐M) to remove Fe, acidified with 0.25% concentrated hydrochloric acid, and purged with a slow stream of N for 10 min to remove dissolved inorganic sulfide. Although all anion samples were acidified, only those with a noticeable colorimetric response for sulfide were purged with N. The entire extraction and preservation process for 12 to 16 wells in a single peeper profile typically took less than 45 min, during most of which the peeper was under a N atmosphere.

### Analytical methods

Sulfate was measured on a Dionex ICS‐1100 ion chromatograph (Thermo Scientific AS22 IonPac 4 × 250–mm anion exchange column) using the Chromeleon software for peak integration. Sulfide and ferrous Fe were quantified spectrophotometrically using the methylene blue method (Eaton et al. 2005a) as implemented with Hach sulfide reagents (method 4500 S2‐D) and the phenanthroline method (Eaton et al. 2005b), respectively. The sulfide method quantifies all dissolved sulfide (both H_2_S and HS^–^) and is therefore not sensitive to pH. Sulfide was quantified within 1 h of collection on a field spectrophotometer, and Fe was stored on ice and transported to the laboratory for quantification within 4 h of collection. A Thermo Orion epoxy pH electrode, with temperature correction calibrated in the field immediately prior to sample measurements was used to measure pH. The detection and reporting limits for each dissolved analyte are summarized in Supplemental Data, Table SI‐1.

Frozen sediment samples were used to quantify acid volatile sulfides (AVSs) immediately after thawing using a strong acid digestion (9 N hydrochloric acid [HCl] + stannous chloride; Eaton et al. 2005a). A separate, homogenized, and split aliquot was used to quantify solid content and loss on ignition as a proxy for C content (Dean 1974). Iron in the solid phase was extracted from freeze‐dried sediment using a weaker acid digestion (0.5 N HCl), as outlined in Myrbo et al. (2017a), and quantified on an Elan 3000 inductively coupled plasma mass spectrometer using a customized standard from Inorganic Ventures. For a subset of sediment samples from the mesocosms, Fe speciation and quantity were investigated using paired weak acid digestions (0.5 N HCl) in which both ferrous and total Fe were quantified with a strong acid digestion (aqua regia) for total Fe. A sequential extraction on the same subset of samples was also used to quantify recalcitrant (pyrite) phases that resist extraction with weaker methods. For these samples, sulfide was extracted sequentially with 0.5 N HCl (paired with Fe speciation [Myrbo et al. 2017a]), 9 N HCl with stannous chloride (Eaton et al. 2005a), and chromium reducible S (Fossing and Jørgensen 1989) and captured for spectrophotometric quantification in 0.2 M zinc actinium using a modified diffusion method (Brouwer and Murphy, 1994) sequentially.

### Data analysis

Depth‐dependent estimates for porosity and bulk density were made from solid content and loss on ignition measurements on sediment samples from intact cores using the relationship outlined in Gosselink et al. (1984). The depth‐integrated mass of S over depth was calculated by integrating the product of concentration and porosity (for dissolved phase) or concentration and bulk density (for solid phase). In most cases, experimental quantities are aggregated over all 4 experimental yr (porewater sulfate mass, porewater sulfide concentration, solid‐phase sulfide) so that each experimental treatment level had 8 replicates during each season (June and August), providing a basis for statistical comparisons. In cases where significant changes in experimental quantities occurred over time (porewater Fe mass, porewater sulfide mass, solid‐phase sulfide), results are presented as the average of duplicate measurements. In these cases, regressions of average measurements versus treatment level or time are used in the interpretation rather than analysis of variance (ANOVA) statistics.

The maximum rate of diffusion‐driven flux was estimated from porewater observations by identifying the largest change in sulfate concentration between adjacent peeper wells and multiplying this gradient by an effective diffusion coefficient by adjusting molecular diffusion for porosity (Boudreau 1996). Significant differences in the main effects of treatment level and year on sulfide and Fe mass in porewater were evaluated using 2‐way ANOVA with repeated measures in SigmaPlot for each season. For sulfate, measurements from all experimental years were aggregated, and the effects of treatment level and season were evaluated using 2‐way ANOVA with repeated measures. Linear regressions using Pearson's correlation test on the average of sulfate porewater mass for all experimental years were used to evaluate the significance of the relationship with overlying water sulfate and time.

The slope of a linear regression of sediment AVS versus year was used to provide an estimate for the rate of annual accumulation of solid‐phase sulfide and to make an estimate for the S:Fe ratio in sediment during years in which sediment cores were not collected. Plant emergence, juvenile survival, filled seed ratio, seed mass, and total biomass data reported in Pastor et al. (2017) were normalized to observations in the control mesocosms during each year to place all observations (from different years) on a consistent basis for comparison to the accumulating solid‐phase and porewater sulfide. In most cases, the mass of sulfate in sediment porewater is expressed in terms of “sulfate as S” (96 mg sulfate = 32 mg sulfate as S) to facilitate stoichiometric comparisons with sulfide quantities in sediment. Surface water sulfate concentrations are expressed “as sulfate” to maintain consistency with prior work on S impacts to wild rice ecosystems (Pastor et al. 2017; Myrbo et al. 2017b).

## RESULTS

### Constant mass of sulfate in porewater

**Figure 1:**
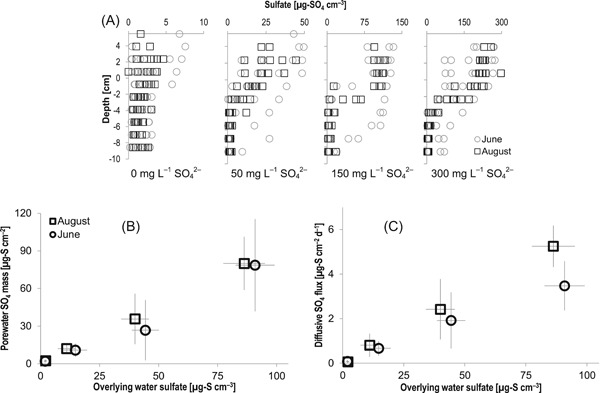
(**A**) Depth profiles of porewater sulfate concentrations (as sulfate) in sulfate‐amended mesocosm sediment in June and August. Data compiled for all years between 2012 and 2015. (**B**) Depth‐integrated porewater sulfate mass (as sulfur) between 0 and 10 cm versus overlying water sulfate (as sulfur). (**C**) Estimated maximum diffusive sulfate mass flux (as sulfur) versus overlying water sulfate (as sulfur). Sulfate labels in (**A**) refer to nominal overlying water concentration (as sulfate) applied during experiments. Vertical error bars in (**B**) and (**C**) represent 1 standard deviation from the mean of 8 replicate measurements for each amendment level, aggregated over 4 experimental yr. Horizontal error bars in (**B**) and (**C**) represent 1 standard deviation from the mean of biweekly measurements of overlying water sulfate concentration during June and August, aggregated over 4 experimental yr.

In porewater near the sediment–water interface, sulfate was consistently elevated at concentrations approaching those present in the overlying water and depleted below 2 to 5 cm (Figure 1A). This pattern unambiguously indicates a supply of sulfate from the overlying water and is consistent with the reduction of sulfate to sulfide in anoxic sediments. Sulfate mass in porewater was strongly correlated with overlying water sulfate concentration in amended mesocosms (Figure 1B; *p* < 0.01, regression coefficient provided in Supplemental Data, Table SI‐2). The mass of sulfate (presented as mass of S) in the top 10 cm of sediment porewater (Figure 1B) ranged from <5 to >75 µg‐S cm^–2^ and did not differ significantly (at each sulfate amendment level) over the course of the 4‐yr experiment (*p* > 0.3 for regression with year; data not shown). Concentrations of sulfate in porewater and surface water were more variable in June, owing to less consistent sulfate amendments and rainwater dilution, though the overall mass of sulfate in porewater was not significantly different in June relative to August (*p* = 0.55).

The observed maximum spatial gradient in porewater sulfate provides an estimate for the maximum diffusive flux of sulfate into sediment. Consistent with the overall mass of sulfate in mesocosm porewater, these estimates for diffusive mass flux of sulfate into sediment (presented as S mass flux) were almost perfectly correlated with overlying water sulfate (Figure 1C; *p* < 0.001, June and August, regression coefficients provided in Supplemental Data, Table SI‐2). Although surface water concentrations were, on average, slightly lower during August (by ~15%), maximum observed sulfate gradients, and hence estimated diffusive fluxes, were significantly (*p* < 0.005) higher in August than June.

### Cumulative increases in porewater sulfide, decreases in porewater Fe

**Figure 2:**
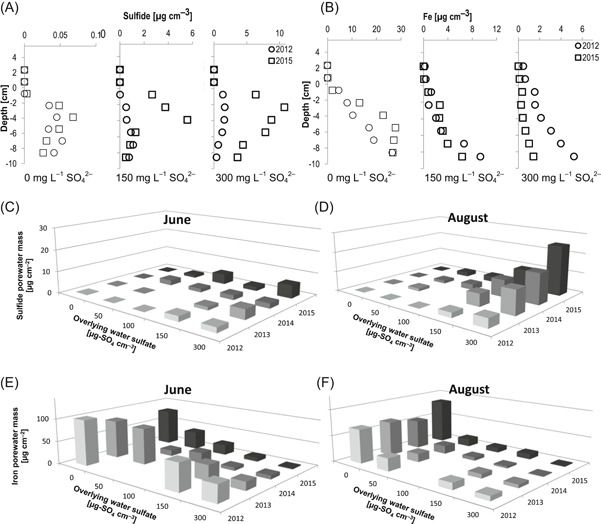
Depth profiles of mean porewater (**A**) sulfide and (**B**) iron in mesocosm sediment at 3 sulfate amendment levels (0, 150, 300 mg L^–1^) in August 2012 and August 2015. Values are the mean of 2 replicate measurements. Depth‐integrated mass of porewater (**C**,**D**) sulfide and (**E**,**F**) iron in sediment between 0 and 10 cm for all sulfate amendment levels during all years in June (**C**,**E**) and August (**D**,**F**). Overlying water sulfate labels refer to nominal overlying water concentration (as sulfate) applied during experiments.

Sulfide concentrations were highest in the top 3 to 5 cm of sediment porewater in all sulfate‐amended tanks at locations coincident with steep spatial gradients in sulfate concentration (Figure 2A). In later years of the experiment, maximum porewater sulfide concentrations exceeded 5 to 10 µg cm^–3^ in tanks amended with 300 mg L^–1^ sulfate in overlying water. Depth‐integrated porewater sulfide mass in the top 10 cm of sediment showed clear patterns both with overlying water sulfate and progressively over the 4‐yr study (Figure 2C and D). During August, porewater sulfide mass increased with overlying water sulfate during every year of the study (regressions included in Supplemental Data, Figure SI‐1). Increases from year to year in porewater sulfide mass were largest during August in the tanks with 150 and 300 mg L^–1^ sulfate in overlying water, but porewater sulfide mass of tanks amended with only 10 and 50 mg L^–1^ sulfate in overlying water also experienced consistent interannual increases in August porewater sulfide mass. During August, depth‐integrated porewater sulfide mass was <0.4 µg cm^–2^ in tanks without sulfate amendment during all years of the experiment, but porewater sulfide mass in sediment subject to the highest sulfate amendments progressively increased to 25 µg cm^–2^ over the 4‐yr experiment.

Dissolved Fe concentration in porewater increased with distance from the sediment–water interface to >25 µg cm^–3^ at depths >5 cm (Figure 2B) in porewaters without sulfate amendment. In mesocosms with >50 mg L^–1^ sulfate in the overlying water, porewater Fe was lower, especially in the surface 5 cm of sediment where sulfide concentrations were elevated. Interannual comparisons showed progressive depletion of depth‐integrated porewater Fe mass, especially in the heavily sulfate‐amended mesocosms (Figure 2E and F). Depth‐integrated porewater Fe mass ranged from 70 to 100 µg cm^–2^ in the tanks without sulfate amendment to 2 to 20 µg cm^–2^ in the tanks with the highest sulfate amendments. The observed patterns showed increases in porewater sulfide mass and decreases in porewater Fe mass both with increasing overlying sulfate and over the course of the 4‐yr study. This strongly suggests that, especially in the tanks receiving the highest sulfate loads, a progressive depletion in the Fe available to complex with dissolved sulfide caused increases in porewater dissolved sulfide.

Seasonal differences in porewater geochemistry are of interest because specific life stages of wild rice appear to be impacted by porewater sulfide. The seedling phase in spring and the seed production phase in late summer are impacted by sulfide, but sulfide only weakly affected the period of vegetation growth in between (Pastor et al. 2017; LaFond‐Hudson et al. 2018). During August, the porewater sulfide mass was 2 to 7 times higher than during June at the highest 2 sulfate treatment levels; however, concentrations in June were more variable and not as consistently related to surface water sulfate (Supplemental Data, Figure SI‐1). Porewater Fe mass was significantly lower during August in all sulfate addition treatments, consistent with the pattern of elevated sulfide during August and the insolubility of Fe–sulfide solid phases. The seasonal differences in porewater sulfide and Fe mass (Figure 2) between June and August suggest that the sediment is experiencing faster rates of sulfate reduction during warmer temperatures in mid‐August (18.5 °C average) relative to early June (11.7 °C average). It is also possible that faster rates of sulfate transport (Figure 1C) enabled faster diffusion under warmer conditions or that the development of sulfate‐reducing biomass led to faster rates of sulfate reduction later in the growing season.

The increases in porewater sulfide mass between June and August (of 10–20 µg cm^–2^ at the highest amendment level) show that inputs of sulfide to sediment porewater are occurring at a (net) rate faster than removal through oxidation or Fe–sulfide precipitation. This is consistent with a progressive increase in oversaturation of FeS in sediment porewater through the summer (ion activity product; Supplemental Data, Figure SI‐2). A more rapid input of dissolved sulfide later in the summer would increase the driving force for FeS precipitation. The consistent relationship between Fe and sulfide in porewater observed during August was likely attributable to the consistent, thermodynamically driven process of FeS precipitation driven by oversaturation. The more variable dissolved Fe and sulfide concentrations in June and the less significant relationships of dissolved sulfide to overlying water sulfate are consistent with less sulfide‐driven thermodynamic constraints on porewater Fe and sulfide when the introduction of sulfide is slower or more variable earlier in the summer.

### Cumulative increases in solid‐phase sulfide

**Figure 3:**
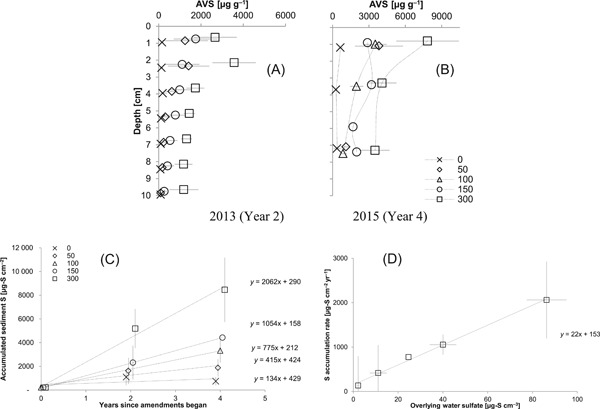
Depth profiles of sediment sulfide concentration (extracted with 9 N HCl + stannous chloride) in sulfate‐amended mesocosm sediment in (**A**) August 2013 and (**B**) 2015. Error bars represent 1 standard deviation from the mean of triplicate measurements at each depth. Depth‐integrated sediment sulfide mass between 0 and 10 cm: (**C**) depth‐integrated mass over 4 yr and (**D**) annual sulfide accumulation rate versus overlying water sulfate. Symbols in (**A**–**C**) refer to nominal overlying water concentration (as mg sulfate L^–1^) applied during experiments. In (**C**) error bars represent 1 standard deviation from the mean of sulfide mass estimates from triplicate depth profiles. In (**D**) vertical error bars represent the 95% confidence interval in the slopes of regressions in (**C**); horizontal error bars represent 1 standard deviation in biweekly measurements of overlying water sulfate concentration during June and August aggregated over 4 experimental yr.

Sulfide in the sediment solid phase (extracted with 9 N HCl) began uniformly low at the outset of the experiment (~80 µg g^–1^, or 212 µg cm^2^) and increased to >4000 µg g^–1^ in surficial sediment after 4 yr in the most heavily sulfate‐amended sediment (Figure 3A and B). Regressions of sediment sulfide against year suggest that, on average, over the first 4 yr of sulfate amendments to mesocosms, sulfide is accumulating in sediment linearly with time (Figure 3C; regression coefficients provided in Supplemental Data, Table SI‐2) and at faster rates in proportion to overlying sulfate concentrations (Figure 3D). Approximately 22 µg cm^–2^ yr^–1^ of sulfide accumulated in the top 10 cm of the sediment solid phase for every µg cm^–3^ of sulfate (as S) present in the overlying water (Figure 3D). Even in experimental mesocosms receiving no sulfate amendments to the overlying water, the sulfate present in the overlying well water (averaging 5–10 mg L^–1^ as sulfate) led to increases in sediment sulfide. In sediment amended with 300 mg L^–1^ sulfate, depth‐integrated sulfide mass in the sediment solid phase exceeded 4000 µg‐S cm^–2^ after only 2 yr of amendment, whereas sediment amended with 150 mg L^–1^ sulfate reached 4000 µg‐S cm^–2^ after 4 yr (Figure 3C).

Sulfate reduction rates in sediment are not typically proportional to sulfate at concentrations above 50 to 200 mg L^–1^ (as sulfate; Pallud and Van Cappellen 2006). However, the linear relationship between solid‐phase sulfide accumulation rate and overlying water sulfate suggests that in these mesocosm sediments the accumulation of sulfide in the solid phase continued to be (at least in part) limited by the supply of sulfate up to approximately 300 mg L^–1^ (as sulfate) in surface waters. In heavily amended mesocosms, the zone of sulfate reduction in sediment may have expanded in response to overlying water sulfate amendments (Figure 1A), thereby creating an opportunity for a net increase in the depth‐integrated (areal) rate of sulfate reduction and Fe sulfide accumulation, even above approximately 100 mg L^–1^ overlying water sulfate.

### Cumulative increase in solid‐phase S:Fe ratio

**Figure 4:**
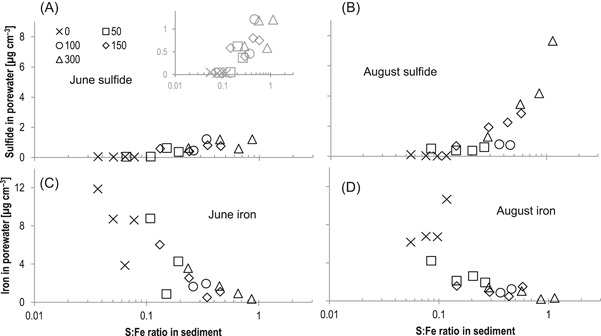
Porewater concentration of (**A**,**B**) sulfide and (**C**,**D**) iron versus the S:Fe molar ratio in sediment during (left) June and (right) August over the course of continuous sulfate loading to sediment in mesocosms. Each point on the graphics corresponds to the average of measurements (top 7 cm of sediment) made during a single season for individual years. Symbols refer to nominal overlying water concentration (as mg sulfate L^–1^) applied during experiments.

Iron concentration in the surface 10 cm of the mesocosm sediment solid phase (extracted with 0.5 M HCl) averaged 144 (±20) µmol g^–1^ (Supplemental Data, Table SI‐4). This equates to a depth‐integrated Fe mass of 305 (±61) µmol‐Fe cm^–2^. Over the course of the 4‐yr study and across a 2–orders of magnitude range in sulfate loading rate, sulfide in sediment porewater began to build up as the stoichiometric quantity of sulfide approached that of Fe in the solid phase (Figure 4A and B). The trend of increasing porewater sulfide with S:Fe ratio was particularly stark during August when average porewater sulfide in the surface 7 cm of sediment ranged from 1 to 8 µg cm^–3^ in mesocosms during years when the S:Fe ratio exceeded 0.2 to 0.5 in the sediment solid phase. In June, concentrations of porewater sulfide were also low (<0.1 µg cm^–3^) in mesocosms with low S:Fe ratios (<0.5), but sulfide increased considerably (>0.5 µg cm^–3^) in mesocosms with S:Fe ratios exceeding 0.2 to 0.5. Analogous trends were present in the relationship between S:Fe ratio and dissolved ferrous Fe (Figure 4C and D). Adverse effects to wild rice have been observed at both the early and late life stages of wild rice's annual life cycle (LaFond‐Hudson et al. 2018). It is, therefore, notable that a similar threshold in S:Fe ratio was related to higher porewater sulfide during both June and August because the processes that control porewater sulfide may differ between seasons.

Although sulfate remained in the overlying water during experiments, Fe was always below detection limits in overlying water (<100 µg L^–1^). An average addition of 178 L yr^–1^ well water with a maximum observed concentration of 0.6 µg cm^–3^ would have increased the extractable Fe content of sediment in mesocosms by <0.4% over the course of the 4‐yr study (18 µg cm^–2^ yr^–1^), assuming all the Fe from the well water went into the 0.5 N extractable phase. On average, >85% of loosely bound (0.5 N HCl) Fe was found to be in the reduced (ferrous) form at all sediment depths (Supplemental Data, Table SI‐4). Assuming a 1:1 stoichiometry in amorphous FeS, approximately 10 000 µg‐S cm^–2^ of sulfide would be needed to bind all extractable Fe into insoluble FeS complexes in the top 10 cm of sediment.

Chromium reduction of sediment S typically extracted <15% additional S from the sediment following 9 N acid extractions in heavily S‐amended tanks. A weaker extraction, using 0.5 N acid, quantified S at levels similar to the 9 N acid (Supplemental Data, Table SI‐4). These sequential extractions suggest that most of the new, solid‐phase inorganic S is present as amorphous or weakly bound monosulfur FeS complexes. For the present analysis, the accumulation of sulfide mass in the sediment solid phase was quantified by normalizing AVS (9 N HCl + stannous chloride) by the pool of ferrous Fe extracted with a 0.5 N acid digestion. Total aqua regia–extractable Fe was 15 to 40% higher than the 0.5 N acid Fe for heavily sulfate‐amended mesocosms and 70 to 120% higher than the 0.5 N acid Fe for control mesocosms (Supplemental Data, Figure SI‐3). Though the exact nature of the pool of solid‐phase Fe and its reactivity toward sulfide was not investigated in detail, the S:Fe ratio chosen for weakly bound FeS (analogous to degree of sulfidation) was used as a means of characterizing the accumulation of S under different loading rates.

### Cumulative impacts on plant growth and reproduction

**Figure 5:**
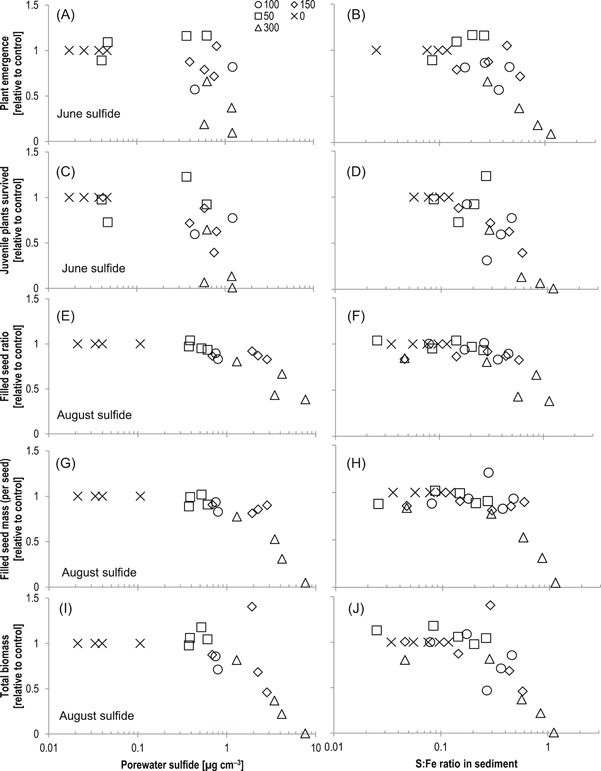
Impacts of sulfide in (left) porewater and (right) sediment solid phase to wild rice reproduction/growth including (**A**,**B**) emergence, (**C**,**D**) juvenile survival, (**E**,**F**) filled seed ratio, (**G**,**H**) filled seed mass, and (**I**,**J**) total biomass. Each point on the graphics corresponds to the average of measurements made at one treatment level during each year. Symbols refer to nominal overlying water concentration (as mg sulfate L^–1^) applied during experiments.

Early in wild rice's annual life cycle, plant emergence and juvenile survival decreased during years when porewater sulfide concentrations during June averaged >0.4 µg cm^–3^ in the top 7 cm of sediment (Figure 5A and C). These results are consistent with the range of sulfide concentrations at which wild rice seedling growth is stunted in hydroponic experiments with wild rice (Pastor et al. 2017) and in the range of those measured for other sensitive freshwater wetland plants (Lamers et al. 2013). These concentrations are well below those reported to be toxic by Fort et al. (2017) in hydroponic experiments. The seedlings in the Fort et al. (2017) experiment (maximum age 21 d) were allowed to emerge into ambient air above the water, whereas the seedlings in the Pastor et al. (2017) experiment (maximum age 17 d) were maintained under water. The greater tolerance of wild rice to sulfide observed by Fort et al. (2017) may be the result of the seedlings’ access to atmospheric oxygen, which would allow the internal detoxification of absorbed sulfide by S dioxygenase (SDO; Krüssel et al. 2014). The activity of SDO is limited by oxygen availability. In nature, 21‐d‐old wild rice seedlings have typically not emerged into the atmosphere (Fort et al. 2017) but are not uniformly subject to sulfide exposure of all tissue (Pastor et al. 2017).

Later in the life cycle, decreases (relative to the control mesocosms) in filled seed ratio, filled seed mass, and total biomass were observed in mesocosms during years when porewater sulfide concentrations during August exceeded 0.7 µg cm^–3^ in the surface 7 cm of sediment (Figure 4E, G, and I). Effects on later life stages of wild rice were observed at higher porewater sulfide concentrations than those known to be toxic to seedlings. However, effects on later life stages may be realized as a result of inhibition of N uptake as Fe sulfide precipitates accumulate on roots and sulfide erodes an oxidized Fe barrier around the root, allowing sulfide to penetrate closer to root surfaces (LaFond‐Hudson et al. 2018).

Across a range of sulfate‐loading rates (<200–2000 µg S cm^–2^ yr^–1^) and times needed to titrate uncomplexed Fe in sediment, the S:Fe ratio was consistently related to biological responses in wild rice plants (Figure 4B, D, F, H, and J). Though effects on wild rice populations observed in June and August occurred when different sulfide levels were present in sediment, molar S:Fe ratios between 0.3 and 0.6 in the sediment solid phase are consistently associated with rapid declines in plant growth and reproductive ability in this mesocosm system with limited inputs of external Fe. This suggests that the S:Fe ratio is important in defining geochemical conditions in sediment that impact wild rice during both spring and summer, even if different thermodynamic and kinetic processes control porewater sulfide in different seasons. Others have suggested that bulk sediment geochemistry is decoupled from wild rice rooting‐zone geochemistry because of radial oxygen loss, root exudates, and consequent microbial niches near and on root surfaces (Jacq et al. 1991; Emerson et al. 1999; LaFond‐Hudson et al. 2018). However, the bulk quantities of Fe and sulfide in the solid phase and porewater provide the boundary condition in which near‐root processes occur, and the solid‐phase S:Fe ratio appears to be an important factor even if near‐root processes introduce additional complexity.

### Porewater sulfide in relation to bulk sediment and surface water

In the experimental mesocosms described in the present study, the sediment solid phase accumulated a net quantity of 1000 to 2000 µg S cm^–2^ yr^–1^ in the highest 2 amendment levels, and a stoichiometrically equivalent quantity of uncomplexed (by sulfide) Fe was bound into AVS (Table 1, rows 1 and 2). However, only 2 to 20 µg S cm^–2^ were present as dissolved sulfide in the sediment porewater during August of the experiment's fourth year, the most sulfidic conditions observed (Table 1, rows 3). Porewater sulfate mass (30–70 µg SO_4_ as S cm^–2^ at the highest 2 amendment levels) was constant over the course of the 4‐yr study but small relative to the quantity of sulfate present in the approximately 23 cm of overlying water of the experimental mesocosms (Table 1, rows 4 and 5). Across more complex, hydrologically dynamic natural settings with a balance between ongoing Fe and S loads, the specific quantities involved will differ; however, in many sulfate‐impacted freshwaters, the relative magnitude of porewater sulfide mass is likely to be much smaller than solid‐phase sulfide mass or mass of sulfate in the overlying water. Although critically important for defining conditions toxic to wild rice and other aquatic plants, porewater sulfide is itself a small phase relative to the ultimate reactants that produce AVS in sediment: sulfate in the overlying water and Fe in the sediment solid phase. In contrast to overlying water sulfate quantity and relatively large cumulative interannual increases in solid‐phase sulfide, the small size of the porewater sulfide pool relative to other phases involved may make it susceptible to the seasonal changes observed in the mesocosms.

**Table 1:** Quantities, accumulation, and fluxes of sulfur (S) in porewater and solid phases in experimental mesocosms

		Overlying water SO_4_	
		50 mg L^–1^	300 mg L^–1^
1	Fe(II) mass in solid phase (µg cm^–2^ [as S])^a^	10 000	10 000
2	S(II) accumulation in solid phase (µg‐S cm^–2^ yr^–1^)	400	2000
3a	S(II) mass in porewater (June) (µg‐S cm^–2^)	1.2	4.5
3b	S(II) mass in porewater (August) (µg‐S cm^–2^)	2.2	18
3c	S(II) accumulation in porewater (June–August)^b^ (µg‐S cm^–2^)	1	13.5
4	SO_4_ mass porewater (June and August) (µg‐S cm^–2^)	8	70
5	SO_4_ mass in overlying water (µg‐S cm^–2^)^c^	390	2300
6a	SO_4_ flux estimated from diffusion (µg‐S cm^–2^ yr^–1^)^d^	150	850
6b	SO_4_ flux potential from advection (µg‐S cm^–2^ yr^–1^)^d^	400	2400

^a^ Mass of Fe (as S) estimated assuming a 1:1 stoichiometry for FeS.

^b^ Assuming 80‐d accumulation between early June and late August.

^c^ For 23‐cm overlying water column.

^d^ Assuming 180 d of ice‐free time conducive to diffusive transport for 0.3 cm d^–1^ advective flow induced by transpiration for a 90‐d growing season.

## Discussion

### Impacts of plants on S accumulation

The maximum sulfate flux into sediment, estimated from diffusion and observed sulfate profiles, ranged from <1 to 5 µg‐S cm^–2^ d^–1^ in sulfate‐amended tanks. If the ice‐free season of approximately 180 d is used as an estimate of the time conducive to sulfate diffusion into and accumulation within sediment, net annual diffusion‐driven sulfate transport to sediment is between 100 and 850 µg‐S cm^–2^ yr^–1^. This is roughly half the observed solid‐phase sulfide accumulation rates. Therefore, an additional transport mechanism besides diffusion is leading to S accumulation in the sediment solid phase. Evapotranspiration could have drawn sulfate‐amended water into the rooting zone and increased the rate of S loading to sediment beyond transport by molecular diffusion. Evaporation rates of 0.2 to 0.6 cm d^–1^ have been found for wetland plants in the region (Lott and Hunt 2001). An evaporation‐driven advective flux of surface water into sediment is alone sufficient to explain the mass accumulation of S in the sediment solid phase (Table 1, rows 6; 0.3 cm d^–1^ × 50 µg‐S as SO_4_ cm^–3^ = 15 µg‐S cm^–2^ d^–1^ for 150 mg sulfate L^–1^ overlying water). The balance between advective and diffusive transport of sulfate into sediment cannot be discerned from our experiments; however, both occur at a rate proportional to overlying water sulfate.

Wild rice and other wetland plants can release oxygen to the rooting zone and sometimes decrease the accumulation of sulfide in sediment (e.g., Koretsky et al. 2008; Myrbo et al. 2017b). Observations in rooted and unrooted locations in the same mesocosms reported by Myrbo et al. (2017b) suggest that the rooting zone of the rice could facilitate subsurface sulfate cycling through the oxidation of AVS in the sediment solid phase. However, interannual increases of solid‐phase S of 200 and 2000 µg‐S cm^–2^ yr^–1^ (Table 1) mean that a net accumulation of S in sediment occurred, even despite some subsurface reoxidation of sulfide. In these mesocosms, plants were thinned to a density that prevented significant competition for nutrients. This limited density may have reduced the ability of plants to oxidize the rhizosphere relative to thicker natural stands.

The net accumulation of between 6 and 13 µg‐S cm^–2^ in sediment porewater between June and August also suggests changes in the balance between sulfide oxidation and sulfate reduction processes over wild rice's annual life cycle (LaFond‐Hudson et al. 2018). Radial oxygen loss should be highest during the portion of the growing season when growth and photosynthesis are greatest. For wild rice, this happens in June and July. Growth and photosynthesis decline in mid‐August when resources in the stems and leaves are transferred to flowers and seeds (Sims et al. 2012). Consistent with this phenology of growth, there was significantly greater porewater sulfide in August compared to June in the mesocosm sediment amended with 150 and 300 mg L^–1^ overlying water sulfate. Changes to plant‐induced oxygen inputs to sediment cannot be easily separated from increased biological activity or more rapid diffusion under warmer August conditions, but both would be expected to result in a shift toward more reducing conditions later in the summer.

### Implications of Fe speciation in sulfate‐impacted sediment

Iron in the solid phase was not quantified each year; however, the observed annual patterns of dissolved Fe and sulfide are consistent with a depletion in the activity of ferrous Fe that has not been sequestered to insoluble and stable Fe sulfide complexes. In some cases the quantity of Fe in sediment porewater could be limited by Fe(III) reduction. However, the relatively small amount of labile (0.5 N) oxidized Fe extracted from sediment (Supplemental Data, Table SI‐4) and Fe(III)’s low solubility under neutral pH conditions (6.2–7.4) suggest that the main control on porewater Fe in the sulfate‐amended mesocosm sediment is the inputs of dissolved sulfide. The solid‐phase Fe measured in the present study (extracted with 0.5 N acid extractable Fe) may not have precisely reflected the total quantity of Fe available to react with sulfide. Indeed, 30 to 120% additional Fe was extracted with aqua regia compared to 0.5 N HCl, suggesting that an additional, more recalcitrant Fe pool is present. The large and consistent increases in porewater sulfide at S:Fe ratios of near 0.5 (based on 0.5 N acid Fe), however, suggest that some Fe extracted with even the weak acid was not available to efficiently remove dissolved sulfide. Reaction rates of solid‐phase Fe with dissolved sulfide can be slow depending on the nature of Fe phases (Wan et al. 2017).

Reduced S was extracted routinely in the present study using 9 N HCl with stannous chloride (Eaton et al. 2005a), a stronger extraction than the 1 N HCl employed in a USEPA draft standard method (Allen et al. 1991). A significant fraction of chromium‐reducible S (20–70%; Supplemental Data, Table SI‐4) was found in sediment from the tanks without sulfate amendment, possibly as a result of aged FeS minerals present in native, unamended sediment. However, little additional S was extracted with chromic acid from mesocosm sediment that had received large experimental sulfate additions, suggesting that most recent S accumulation was in a labile phase accessed by both 0.5 and 9 N acid extractions (Supplemental Data, Table SI‐4). The nature of the Fe pools present in natural settings would need to be investigated across a wider variety of geologic and hydrologic conditions and utilizing a larger suite of extraction methods to understand the significance of the thresholds of S:Fe ratios that lead to increased porewater sulfide in the mesocosm conditions of the present study.

### Implications of S accumulation in natural systems

The nearly static quantity of Fe in the present study provides a convenient and tractable system to describe the impacts of a change in S loading on porewater geochemistry. Natural settings, however, have ongoing Fe loads from surface water and groundwater that compete stoichiometrically with ongoing S loads to determine the net S:Fe ratio in surficial sediment. A more thorough analysis, via modeling or detailed observations in a variety of S‐ and Fe‐loading scenarios, would be needed to constrain the importance of ongoing sulfate loads, Fe loads, and sediment accumulation rates in determining S:Fe ratios in a natural setting. The quantity of sulfide in the solid phase (AVS) was highest in surface 0‐ to 5‐cm sediment at all sulfate amendment levels, showing that sulfide accumulates most rapidly in the surficial sediment where sulfate reduction is most rapid. Because sulfate was almost completely depleted at depths >5 cm, the elevated AVS concentrations at depths <5 cm suggest that significant mixing of surficial sediment with sediment from lower depths occurred in these mesocosms. The present analysis integrated S and Fe mass over the top 10 cm of sediment to facilitate consistent stoichiometric comparisons in the simplified mesocosm system. However, there were variations in the S:Fe ratio over the top 10 cm of sediment, and the distribution in a natural setting would depend on the source of Fe and S to surficial sediment (groundwater vs surface water). In addition, the relevant depth for assessing S:Fe ratios and porewater sulfide could be different for different wetland plants. The results observed in the present simplified system are useful to understand how porewater geochemistry evolved over time in the context of a range of loading rates and accumulations of S mass in sediment. However, in natural settings the specific quantities, net loading rates, and times related to porewater sulfide accumulation and biological effects are likely to differ from these controlled observations.

In a broad survey of sediment chemistry in natural wild rice stands, sediment C, surface water sulfate, and sediment extractable Fe (0.5 N HCl) were used to predict porewater sulfide (Pollman et al. 2017). The inclusion of solid‐phase AVS (or the difference between extractable Fe and AVS) did not improve the model performance appreciably. The Pollman et al. (2017) field data set was composed mostly of freshwater sediment from ecosystems relatively low in sulfate, with consequently low S:Fe ratios in sediment. In a large data set with typically great stoichiometric excess of Fe over sulfide, it is understandable that the quantity of sulfide in sediment did not rise to the top as an important variable. However, an analysis that included only sediments with relatively large S:Fe ratios may conclude that sediment AVS, in addition to sediment Fe, is consequential in controlling porewater sulfide. An analysis of, for instance, the upper quartile of sediment S:Fe ratios would be especially relevant because these sites are likely the ones most at risk of experiencing a buildup in porewater sulfide in response to additional sulfate loads.

The observation that sulfate did not increase in sediment porewater over the course of the experiments suggests that in this system sufficient C and Fe were both present in sediment to continue driving the reduction of sulfate to sulfide and the removal of sulfide from the dissolved phase, thereby maintaining the flow of sulfate from the overlying water. If C had become limiting before Fe, sulfide would continue to be removed from porewater by excess Fe and an increase in porewater sulfate would be expected as a result of continued diffusion and a lack of electron donors to drive the reduction of porewater sulfate. If Fe had become limiting before C, porewater sulfide would be expected to build up in the absence of a process to remove it from solution and lower its activity. Eventually, porewater sulfide would increase to levels sufficient to cause upward diffusion and oxidation of sulfide near the oxidized sediment–water interface. In this case of excess C relative to Fe, a finite pool of S could continue to cycle in the dissolved phase of the sediment between sulfate near the surface and sulfide at deeper depths.

## CONCLUSIONS

The sediment Fe buffer against a buildup in dissolved sulfide during continuous sulfate loading was overwhelmed relatively quickly (2–4 yr) in experimental mesocosms with 300 and 150 mg L^–1^ sulfate in overlying water. Across a range of sulfate‐loading rates, conditions in sediment porewater shifted from Fe‐rich (>2–5 µg cm^–3^) toward sulfidic (>0.5–2 µg cm^–3^) as S:Fe ratios (as defined by the weak acid extract used in the present study) approached 0.5. Though different sulfide concentrations were present in June and August, S:Fe ratios approaching 0.5 were consistently associated with effects in both early and late stages of wild rice's annual growth and reproductive life cycle. The quantity of uncomplexed Fe (low S:Fe ratio) appears to be important in defining porewater sulfide and consequent effects on wild rice under both spring and summer conditions.

The net S loading rates to the sediment (<200 to >2000 µg‐S cm^–2^ yr^–1^) across the overlying water sulfate amendments in the present study (8–300 mg L^–1^ as sulfate) were proportional to overlying water sulfate. Approximately 22 µg‐S cm^−2^ yr^–1^ accumulated in the sediment solid phase for every µg of S per cm^–3^ of sulfate (as S) present in the overlying water. Eventually, in the absence of significant external Fe inputs, the supply of Fe in the tanks with <10 and 50 mg L^–1^ sulfate in the overlying water would be expected to be overwhelmed by external sulfate loads. The simple linear trends for sulfide accumulation rate in this experiment suggest that this might occur in 10 to 35 yr for the 50 and approximately 10 mg L^–1^ overlying water sulfate, respectively. Analyses of natural systems, however, should consider external loads of Fe, the potential for oxidation of sulfide, and rates of sediment burial (or mobility) to constrain the time frame of impacts attributable to an increase in S loading. In addition, efforts to predict dissolved sulfide from bulk sediment and water parameters should account for the fact that, although ultimately driven by the quantities of Fe, S, and C, the concentration of porewater sulfide is a relatively small phase, potentially subject to seasonal variability, compared with solid‐phase Fe and S.

The results from the present mesocosms are not entirely equivalent to natural settings with ongoing loads of Fe and complex hydrologic conditions. They do, however, provide an opportunity to examine in detail some of the mechanisms involved in the dynamic relationship among surface water chemistry, sediment geochemistry, and the health and reproduction of sensitive freshwater organisms following a change in S loading. In addition to toxicity to wild rice, many other aquatic species are sensitive to sulfide (Kinsman‐Costello et al. 2015), and the present results could help us to understand and develop management strategies to alleviate the ecological effects of increased S loading (Geurts et al. 2009; Greaver et al. 2012). The results illuminate more fully an evolving geochemical setting in which cumulative S loading over a 4‐yr mesocosm study resulted in a wholesale shift from Fe‐dominated to sulfide‐dominated porewater chemistry. This shift was accompanied by detrimental effects to and eventually extirpation of self‐perpetuating wild rice populations.

## Supplemental Data

The Supplemental Data are available on the Wiley Online Library at DOI: 10.1002/etc.4410.

## Disclaimer

The statements, findings, conclusions, and recommendations are those of the author(s) and do not necessarily reflect the views of Fond du Lac, Minnesota Pollution Control Agency, NOAA, the Sea Grant College Program, or the US Department of Commerce.

## Supporting information

This article contains online‐only Supplemental Data.

Supporting information.

## Data Availability

The present study contains supplemental online material. Readers interested in data can contact N. Johnson (nwjohnso@d.umn.edu).
