# Supplementary material for: Cumulative Sulfate Loads Shift Porewater to Sulfidic Conditions in Freshwater Wetland Sediment
Source: Environ Toxicol Chem. 2019 May 27;38(6):1231–44. doi: 10.1002/etc.4410 (PMC6852076; doi:10.1002/etc.4410)
Supplement: Supplementary file 1 — Supporting information. [file ETC-38-1231-s001.docx]

**SUPPLEMENTARY ONLINE MATERIAL**

**Table SI-1** Methods, reporting units, and detection and reporting limits for chemical analyses of porewater.

|  | Units | Method | Detection Limit | Reporting Limit |
| --- | --- | --- | --- | --- |
| Iron (ferrous) | micromoles / liter | Phenanthroline spectrophotometry | 2.78 | 5.05 |
| Sulfide | micromoles / liter | Hach spectrophotometry (methylene blue) | 0.69 | 1.89 |
| pH | -log[H+] (moles) | glass / epoxy pH electrode |  |  |
| Sulfate | milligrams / liter | Ion chromatography | 0.11 | 0.20 |

Figure SI-1 Regression of depth-integrated porewater sulfide mass against (left) year and (right) overlying water sulfate concentration during (top) June and (bottom) August. Regression equations and coefficients are provided in order of decreasing sulfate amendment (left) or year (right).

**Figure SI-2** Seasonal variations in ion activity product for FeS in in surface 5 cm of mesocosm porewaters during 2013 (year 2 of experiment).

**Table SI-2** Regression statistics for correlations of sediment porewater sulfate and maximum diffusive flux vs. overlying water sulfate.

| Depth [cm] | slope | intercept | R^2^ | P |
| --- | --- | --- | --- | --- |
| Porewater SO4 mass (Fig 1b) | 0.89 | -1.5 | 0.98 | < 0.01 |
| Diffusive SO4 flux (Fig 1c, Jun) | 0.038 | 0.094 | 0.99 | < 0.01 |
| Diffusive SO4 flux (Fig 1c, Aug) | 0.061 | 0.020 | 0.99 | < 0.01 |
| sediment S accumulation (Fig 3c, 300 ppm) | 2,092 | 290 | 0.98 | < 0.01 |
| sediment S accumulation (Fig 3c, 150 ppm) | 1,054 | 158 | 0.99 | < 0.01 |
| sediment S accumulation (Fig 3c, 100 ppm) | 775 | 212 | N/A | N/A |
| sediment S accumulation (Fig 3c, 50 ppm) | 414 | 424 | 0.97 | < 0.05 |
| sediment S accumulation (Fig 3c, 0 ppm) | 134 | 429 | 0.65 | < 0.05 |
| sediment S accumulation (Fig 3d) | 22 | 153 | 0.99 | < 0.01 |

**Table SI-2** Fe [umol g^-1^] quantified in 0.5 N HCl in replicate mesocosms at 4 sulfate treatment levels. This data is used to quantify Fe in sediment for normalizing accumulating S in sediment.

| Depth [cm] | 0 mg/L SO4 | 50 mg/L SO4 | 150 mg/L SO4 | 300 mg/L SO4 |
| --- | --- | --- | --- | --- |
| 0.78 | 184 (+/- 49) (n=10) | 198 (+/- 37) (n=9) | 167 (+/- 29) (n=11) | 182 (+/- 39) (n=10) |
| 2.34 | 154 (+/- 46) (n=10) | 161 (+/- 23) (n=10) | 147 (+/- 29) (n=11) | 154 (+/- 26) (n=11) |
| 3.9 | 162 (+/- 87) (n=10) | 139 (+/- 18) (n=9) | 134 (+/- 40) (n=11) | 145 (+/- 29) (n=10) |
| 5.46 | 130 (+/- 21) (n=10) | 144 (+/- 20) (n=9) | 126 (+/- 40) (n=11) | 138 (+/- 25) (n=10) |
| 7.02 | 133 (+/- 26) (n=10) | 135 (+/- 34) (n=10) | 125 (+/- 43) (n=11) | 132 (+/- 36) (n=10) |
| 8.58 | 132 (+/- 34) (n=11) | 139 (+/- 30) (n=9) | 117 (+/- 40) (n=12) | 136 (+/- 35) (n=10) |
| 10.14 | 131 (+/- 25) (n=8) | 142 (+/- 22) (n=9) | 125 (+/- 39) (n=10) | 129 (+/- 38) (n=9) |

Table SI-4 Fe quantified in 0.5 N HCl (ferrous and total in the same extraction procedure) and aqua regia (separate extraction). Results from a sequential sulfide extraction on a subset of sediments are presented as incremental reduced S extracted from 0.5 N HCl, 9 N HCl with stannous chloride, and chromic acid.

|  | | | Sulfide [µmol g^-1^] | | | | Iron [µmol g^-1^] | | | | | Physical Properties | | | | |
| --- | --- | --- | --- | --- | --- | --- | --- | --- | --- | --- | --- | --- | --- | --- | --- | --- |
|  |  |  |  |  |  |  | 0.5 N HCl | | | aqua regia | |  |  |  |  |  |
| Treatment level [mg_-SO4_ L^-1^] | Tank # | Depth [cm] | 0.5 N HCl fraction | 9 N HCl fraction | CRS fraction | (0.5 N+9 N) / CRS | Fe(II) | Fe(tot) | Fe(II) / Fe(tot) in 0.5 N HCl | Fe(tot) | 0.5 N Fe(tot) / Aqua Regia (tot) | % solid | LOI [%] | Est. Carbon [%] | Bulk dens. [g cm^-3^] | Por-osity |
|  |  |  |  |  |  |  |  |  |  |  |  |  |  |  |  |  |
| 0 | 4 | 0-1 | 3.9 | 0.4 | 6.3 | 40% | 160 | 176 | 91% | 274 | 64% | 0.21 | 25 | 12 | 0.21 | 0.40 |
| 0 | 4 | 4-6 | 1.4 | 0.5 | 6.9 | 22% | 131 | 121 | 108% | 329 | 37% | 0.29 | 20 | 10 | 0.26 | 0.36 |
| 0 | 25 | 0-1 | 5.7 | 0.9 | 1.4 | 82% | 177 | 198 | 89% | 308 | 64% | 0.18 | 27 | 14 | 0.19 | 0.42 |
| 0 | 25 | 1-2 | 11.5 | 1.0 | 7.4 | 63% | 159 | 165 | 96% | 291 | 57% | 0.16 | 30 | 15 | 0.17 | 0.44 |
| 0 | 25 | 2-3 | 4.0 | 0.9 | 0.1 | 99% | 131 | 154 | 85% | 230 | 67% | 0.21 | 28 | 14 | 0.19 | 0.38 |
| 0 | 25 | 3-4 | 3.8 | 0.6 | 8.9 | 33% | 139 | 148 | 94% | 266 | 56% | 0.21 | 28 | 14 | 0.19 | 0.38 |
| 0 | 25 | 4-6 | 4.0 | 0.7 | 3.4 | 58% | 134 | 157 | 85% | 292 | 54% | 0.22 | 26 | 13 | 0.20 | 0.38 |
| 0 | 25 | 6-10 | 2.8 | 0.4 | 5.4 | 38% | 137 | 149 | 92% |  |  | 0.27 | 24 | 12 | 0.22 | 0.34 |
| 100 | 26 | 0-1 | 39.8 | 3.1 | 11.5 | 79% | 109 | 115 | 95% | 212 | 54% | 0.25 | 22 | 11 | 0.24 | 0.39 |
| 100 | 26 | 1-2 | 43.0 | 3.6 | 18.9 | 71% | 127 | 132 | 96% | 242 | 55% | 0.23 | 26 | 13 | 0.20 | 0.36 |
| 300 | 17 | 0-1 | 143.3 | 5.2 | 29.6 | 83% | 231 | 203 | 114% | 264 | 77% | 0.15 | 29 | 14 | 0.18 | 0.48 |
| 300 | 17 | 1-2 | 179.7 | 1.2 | 4.5 | 98% | 213 | 204 | 104% | 255 | 80% | 0.18 | 27 | 13 | 0.19 | 0.44 |
| 300 | 17 | 2-3 | 101.2 | 5.5 | 9.0 | 92% | 184 | 185 | 99% | 240 | 77% | 0.23 | 25 | 12 | 0.21 | 0.38 |
| 300 | 17 | 3-4 | 99.1 | 3.4 | 13.6 | 88% | 147 | 140 | 105% | 315 | 44% | 0.26 | 22 | 11 | 0.24 | 0.37 |
| 300 | 17 | 6-10 | 43.2 | 1.2 | 6.1 | 88% | 142 | 144 | 99% | 204 | 71% | 0.27 | 25 | 12 | 0.21 | 0.33 |
| 300 | 19 | 0-1 | 39.1 | 1.1 | 19.5 | 67% | 128 | 125 | 102% | 176 | 71% | 0.20 | 22 | 11 | 0.24 | 0.46 |

**Figure SI-3** Incremental fractions of solid-phase Fe extracted from sediment in wild rice mesocosms. Tank numbers are included with sulfate amendment level in the labels.
